# Supplementary material for: Gluten Degradation by the Gut Microbiota of Ulcerative Colitis Patients
Source: Microorganisms. 2022 Dec 21;11(1):12. doi: 10.3390/microorganisms11010012 (PMC9867242; doi:10.3390/microorganisms11010012)
Supplement: Supplementary file 1 [file microorganisms-11-00012-s001.zip › microorganisms-2070272-supplementary.pdf]

Supplementary Materials:

Characteristics	UC	Healthy subject	P value
**n (%)**	12 (48)	13 (52)	
**Age, y (SD)**	44.33 (13.28)	38.69 (15.98)	0.35
**Sex (%)**			
Female	6 (50)	5 (38,5)	
Male	6 (50)	8 (61,5)	
**Race (%)**			
White	8 (66.6)	10 (76)	
Other	4 (33.3)	3 (23)	
**Smoker (%)**	0 (0)	0 (0)	1
**Anthropometrics**			
Height, in (SD)	66.67 (3.6)	68.77 (4.45)	0.21
Weight, kg (SD)	70.34 (14.73)	71.76 (11.61)	0.78
BMI, kg/m2 (SD)	24.5 (4.8)	23.51 (2.8)	0.53

Supplemental Table S1. Patient characteristics. UC= ulcerative colitis, SD= standard deviation.

Variable	P-value
Daily servings of bread, cereals, rice, pasta	0.457
Cooked cereal	0.967
Cold cereal	> 0.999
Pizza	0.599
Macaroni and cheese	0.724
Noodles	0.529
Biscuits	0.203
Burger rolls	0.949
Bagels and English muffins	0.986
Flour tortillas	0.815
White bread	0.364
Chips	0.967
Crackers	0.373
Donuts	0.870
Cakes	0.513
Cookies	0.675
Pie	0.177
Beer	0.281

Supplemental Table S2. Food items containing gluten from the Block 2005 Food Frequency Questionnaire, p-values obtained using Mann-Whitney test.

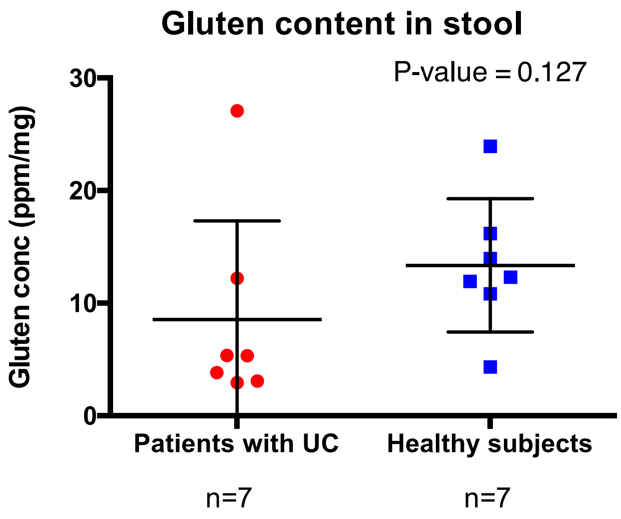

Supplemental Figure S1. Repeated ELISA measuring undegraded gluten in stool samples from patients with ulcerative colitis and healthy subjects. P-value obtained using Mann-Whitney test.

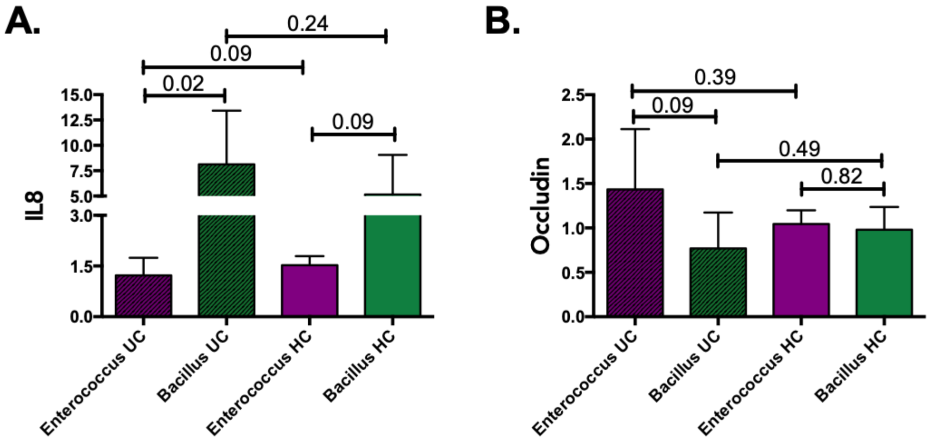

Supplemental Figure S2. Expression of A) IL8 and B) occludin genes in colonic epithelial cells exposed to gluten degradation products produced by Bacillus and Enterococcus isolates from UC and HC subjects. Results were obtained from two independent experiments using three biological replicates. Statistical significance was determined using Mann-Whitney test.
